# New Appliances and Things Medical

**Published:** 1900-04-07

**Authors:** 


### NEW APPLIANCES AND THINGS MEDICAL.

**[We shall be glad to receive, at our Office, 28 & 29, Southampton Street, Strand, London, W.C., from the manufacturers, specimens of all new preparations and appliances which may be brought out from time to time.]**

### CARNOS.

(Carnos Limited, Wragby Street Works, Great Grimsby.)

Carnos is a concentrated fluid extract, of which the average analysis, according to the manufacturers' report, is as follows:—

| | |
|---|---|
| Albuminoids | 22·28 |
| Carbohydrates | 15·31 |
| Phosphoric Acid | 3·41 |
| Mineral matter | 1·29 |
| Potash | 3·30 |
| Moisture | 54·41 |

An examination of the above figures shows that, when compared to the usual meat extracts, the presence of carbohydrates to the extent of 15 per cent. removes it altogether from this class of nitrogenous foods, although possessed to a certain extent of the same properties it has in addition a carbohydrate or energy-producing value, and hence, though invigorating to the nerves and other tissues, it has sustaining properties that are not to be met with in the usual meat extracts. Carnos is decidedly pleasant to the taste, and deserves to be appreciated not only in the sick room but also by strong and vigorous persons whose habits of life expose them to occasional spells of severe strain, whether mental or bodily. For athletes and brain workers Carnos is particularly suited. The rapidity with which it is absorbed into the system enables the consumer to experience the benefits which it confers almost immediately after taking.

### THERMOGENE.

(Fasset and Johnson, 31 and 32, Snow Hill, London, E.C.)

Thermogene is a revulsory and resolvent wadding—that is to say, it is a wool impregnated with a revulsive which, when applied to the skin, keeps up a continual and constant revulsive action. Unlike the ordinary blistering preparation it causes no pain or disagreeable sensation, but maintains a sustained thermogenic effect upon the skin. In cases of neuralgia and rheumatism, if the affected part be wrapped in the wadding, there is an agreeable sense of warmth and almost immediate mitigation of the pain. We have had personal experience of its efficacy in cases of rheumatic inflammation of the knees and obstinate lumbago; the patients have one and all expressed their appreciation of the wool. A note of warning may be given against shaking out the wool in proximity with the mouth, eyes, or nose; the irritating properties of the impregnated drug, though grateful and comforting to the integuments, is anything but soothing to the more sensitive and tender mucous membranes.

### PATENT COOKED OATMEAL.

(George King and Co., Albion Food Mills, Sycamore Street, London, E.C.)

Oatmeal, as everybody knows, constitutes an excellent breakfast dish, but it does not find the same popularity with the cook because it takes a long time to make properly, and this necessitates early rising. For this reason they discontinue to provide it on the smallest excuse. King's patent cooked oatmeal requires one minute's boiling, and hence is one of the easiest and quickest cooked of all breakfast dishes. Being scientifically prepared by the manufacturers nothing is left to the incompetence of the cook, the porridge is always the same, and always sufficiently boiled, and hence it may be relied upon as being digestible and nutritious. The oatmeal is supplied in carefully sealed tins for threepence each. It may be had fine or medium, is concentrated and compressed, and hence is economical.

### SHREDDED WHEAT BISCUIT.

(Shredded Wheat Company, St. George's House, 6 and 8, Eastcheap, London, E.C.)

Shredded wheat is an ingeniously prepared and cooked whole wheat. The grain after careful washing is boiled by a special process and torn into shreds by appropriate means. The shredded mass is subsequently rolled and cut into pieces of a convenient size. These are then baked and then rebaked. The whole process is exceedingly cleanly, every detail being carried out by machinery. The completed biscuit is particularly nice to eat as it is; it may, however, be converted into toast, or made use of according to some hundreds of recipes—in fact, it can be used almost wherever flour or bread are used. The purity of the preparation and the careful method of production give it a great advantage over these two usual household necessities from the point of view of health. Shredded wheat biscuit consists of nothing but whole wheat, and nothing has been removed. Those who are interested in household and invalid cookery should write for the little pamphlet entitled "The Vital Question," which is published by the Shredded Wheat Company, 6 and 8, Eastcheap, E.C. It contains a great number of useful recipes of various breakfast, lunch, and dinner dishes in which shredded wheat biscuits are employed.

